# Evaluation of a digital patient education programme in patients with coronary artery disease, a survey-based study

**DOI:** 10.1186/s12913-024-11374-5

**Published:** 2024-09-02

**Authors:** Anna Sandberg, Annica Ravn-Fischer, Annika Johnsson, Maria Lachonius, Maria Bäck

**Affiliations:** 1 Institute of Medicine, Department of Molecular and Clinical Medicine, Sahlgrenska Academy, University of Gothenburg, Gothenburg, SE-405 30 Sweden; 2 Department of Occupational Therapy and Physiotherapy, Sahlgrenska University Hospital, Gothenburg, Sweden; 3 Center for Digital Health, Sahlgrenska University Hospital, Gothenburg, Sweden; 4 Department of Cardiology, Sahlgrenska University Hospital, Gothenburg, Sweden

**Keywords:** Cardiovascular disease, Digital technologies, Distance education, eHealth, Health care surveys, Health education, Secondary prevention, Telemedicine, Usability testing

## Abstract

**Introduction:**

Patient education programmes focusing on risk factor modification and lifestyle changes are well established as part of cardiac rehabilitation in patients with coronary artery disease (CAD). As participation rates are low, digital patient education programmes (DPE) are interesting alternatives to increase access. Understanding patients’ perceptions of DPE are important in terms of successful implementation in clinical practice but are not well known. Therefore, the aim of this study was to assess patients’ perceptions of using a DPE in terms of end-user acceptance and usability, perceived significance for lifestyle changes and secondary preventive goal fulfilment in patients with CAD.

**Methods:**

This was a cross-sectional survey-based study. The survey was distributed to all 1625 patients with acute coronary syndrome or chronic CAD with revascularisation, who were registered users of the DPE between 2020 and 2022 as part of cardiac rehabilitation. The survey contained 64 questions about e.g., acceptance and usability, perceived significance for making lifestyle changes and secondary preventive goal fulfilment. Patients who had never logged in to the DPE received questions about their reasons for not logging in. Data were analysed descriptively.

**Results:**

A total of 366 patients (mean age: 69.1 ± 11.3 years, 20% female) completed the survey and among those 207 patients (57%) had used the DPE. Patients reported that the DPE was simple to use (80%) and improved access to healthcare (67–75%). A total of 69% of the patients were generally satisfied with the DPE, > 60% reported that the DPE increased their knowledge about secondary preventive treatment goals and approximately 60% reported having a healthy lifestyle today. On the other hand, 35% of the patients would have preferred a hospital-based education programme. Among the 159 patients (43%) who had never used the DPE, the most reported reason was a perceived need for more information about how to use the DPE (52%).

**Conclusions:**

This study shows an overall high level of patient acceptance and usability of the DPE, which supports its continued development and long-term role in cardiac rehabilitation in patients with CAD. Future studies should assess associations between participation in the DPE and clinical outcomes, such as secondary preventive goal fulfilment and hospitalisation.

**Supplementary Information:**

The online version contains supplementary material available at 10.1186/s12913-024-11374-5.

## Introduction

As early mortality for patients with acute coronary artery disease (CAD) has declined in recent years, there is a large need for effective secondary prevention strategies to further improve long-term prognosis [1, 2]. Secondary prevention provided through comprehensive cardiac rehabilitation (CR) programmes is essential to reduce the risk of mortality, recurrent cardiovascular events, and to improve health-related quality of life [3, 4]. As CR has also been found to be cost-effective [5], participation has received the highest class of recommendation and level of evidence in European guidelines and should be offered to all patients with a diagnosis of CAD [6]. Multidisciplinary CR is a complex intervention that should include individual patient assessment, management and control of cardiovascular risk factors, physical activity counselling, exercise training prescription, dietary advice, psychosocial management, vocational support, and lifestyle behaviour change including patient adherence and self-management [7].

Patient education programmes, as part of CR, can be defined as a planned, and patient-centered intervention that focuses on treatment, risk factor modification, and lifestyle change [8]. A central part of such programmes is to promote self-care behaviour and to encourage patients to take an active part in managing their disease, aiming to improve health outcomes [9]. Education programmes for patients with CAD have been shown to improve disease-related knowledge, self-reported health behaviours, and psychological outcomes [8, 10, 11], but with no clear effects on hospitalisations and mortality [10].

Despite the beneficial effects of centre-based CR programmes, participation rates are low [2, 12]. Well-known barriers for CR-participation include e.g. a long distance to the hospital, transportation difficulties [13] and most recently the Covid-19 pandemic likely caused most centre-based CR programmes to temporarily close down. These challenges have increased the need to develop more accessible modes of CR delivery, such as digital health interventions [14]. Some studies have indicated that for the secondary prevention of CAD, digital patient education programmes (DPE) may have positive effects on psychosocial outcomes, modifiable risk factors and to increase patient knowledge and satisfaction [15, 16]. There is, however, a large heterogeneity across modes of delivery and various outcomes and additionally the patients’ perceptions of DPE are not well-known [15, 17, 18]. To understand patient acceptance and usability of digital health systems is crucial for a successful implementation into clinical practice [17, 18]. Therefore, the aim of this study was to assess the perceptions of DPE as part of CR, in terms of end-user acceptance and usability, perceived significance for lifestyle changes and secondary preventive goal fulfilment in patients with CAD.

## Materials and methods

### Design

This is a cross-sectional survey-based study.

### The digital patient education programme

The DPE was developed in 1177, the Swedish national platform for developing digital support and treatment programmes, as an alternative or adjunct to the usual care centre-based education programme. The developers consisted of participants from the CR team with multi-professional competence as well as patients and their relatives. The design of the DPE was performed in an iterative process, in co-design with the end-users. First topics and content of the DPE was identified based on previous studies and user group sessions. Then a prototype of the DPE was developed, which was refined and finalized, including a paper prototype followed by testing and approval of the digital version of the DPE [19].

The DPE covers similar core content as the centre-based education programme, targeting the secondary prevention of CAD, including information about the disease and treatment, risk factors, medications, emotional responses, lifestyle recommendations such as physical activity and exercise, healthy food choices, tobacco and alcohol use. The DPE also includes education modules about the follow-up visits to the out-patient CR centre and information from the Heart- and Lung Association. Overall, the DPE consists of 13 modules including written information, short video clips and illustrations. There are also interactive functions with opportunities for patients and healthcare professionals to send messages and for patients to fill in a questionnaire (Alcohol Use Disorders Identification Test, AUDIT), the results of which are shared digitally with the healthcare professionals. Patients need to have access to the internet, basic computer skills and they are required to have a digital identification to log on to the 1177 platform. The information in the DPE is only available in Swedish. Patients had access to the DPE for 1 year.

### Participants and data collection procedure

As part of usual care, patients with a diagnosis of CAD, including acute coronary syndromes or chronic CAD with percutaneous coronary intervention or after coronary artery bypass grafting are offered participation in an education programme as a standard component of CR. CR is provided as an out-patient programme, however, patients got access to the DPE before discharge from hospital. Participation in the DPE was followed-up during regular visits to the outpatient CR-centre after a few weeks. The DPE was launched in April 2020 and, during the Covid-19 pandemic when the centre-based education programme was put on hold, the DPE was the only available education programme alternative. All eligible patients with CAD who were registered users in the DPE between April 2020 and June 2022 at the Sahlgrenska University Hospital were contacted by mail with an invitation to participate in the study. Patients who wanted to participate in the study could answer the survey in two ways: (1) By answering the paper survey and return it together with the written consent form in a pre-paid envelope to the researchers, or (2) By answering the survey digitally and in those cases, patients gave digital informed consent. Non-responders did not receive any reminder. The study was approved by the Swedish Ethical Review Board (registration number 2022-01783-01).

### Survey design and content

The survey was designed by a research team consisting of physiotherapists, nurses, and cardiologists, all with extensive clinical experience in CR, and one representative with expertise in developing support and treatment programmes at the 1177 platform.

The aim of the survey was to assess patients’ perceptions among both patients who had experience with using the DPE, and those who received access to the DPE but never logged in. Therefore, the first question asked whether the patient had logged in to the DPE (yes/no). Patients who had never logged in, received one follow-up question with predetermined reasons for not logging in and the possibility to add further reasons in free text. Patients who had logged in to the DPE received 64 questions in total about if they had completed the different DPE modules, if they found the modules interesting and the perceived significance for making lifestyle changes and secondary preventive goal fulfilment after completing the DPE. The last question provided an opportunity for patients to share additional experiences or opinions about the DPE in free text.

The survey included questions concerning *patients’ acceptance of using the DPE* (3 questions). Those questions were designed based on clinical experience and evaluated whether patients would have preferred the traditional centre-based programme over the DPE. The survey’s questions concerning *usability* was inspired by the Telehealth Usability Questionnaire (TUQ) that evaluates the usability of telehealth services and implementation [19]. Attributes of usability include six different factors and the factor description similar to Parmanto et al. was used, to ensure internal and external validity of our instrument [19]:

1. *Usefulness* (4 questions) refers to the patients’ perception of how the DPE functions to provide patient education similar to the traditional centre-based programme.
2. *Ease of use and learnability* (2 questions) deals with if the DPE was simple to use and if it was easy to learn how to use.
3. *Interface quality* (3 questions) handles with how pleasant the DPE was to use for the patient.
4. *Interaction quality* (3 questions) measures the patient’s interaction with the clinician and these questions were modified to suit the DPE (i.e. written information and videos).
5. *Reliability* (1 question) refers to if the DPE is as reliable as an in-person visit.
6. *Satisfaction and future use* (4 questions) are related to overall patient satisfaction with the DPE and how willing the patient would be to use the DPE in the future.

Prior to distribution, content validity of the survey was tested by asking five experienced healthcare professionals to provide feedback and to address whether the questions measure what they intend to. They also provided feedback on whether the survey seemed to have all the questions needed for the purpose of the study. Based on their comments some of the questions were slightly modified. Face validity was tested by interviewing five patients with CAD on how they perceived the questions in the survey. No modifications needed to be made. The survey is available as a supplementary file.

### Data analysis

Demographic characteristics and survey results were presented descriptively. Continuous variables were presented with the mean and standard deviation and categorical variables were presented using frequencies and percentages. The survey mainly consisted of grading scales and in some cases, the patients were able to write a free text answer when the given answers were not applicable or to provide additional information. For the five-level grading scale questions, the patients were asked to agree or disagree on a scale from “completely disagree” to “completely agree”. For this study, answering “Completely agree” and “Strongly agree” were combined to form the category “Agree” while answering, “Completely disagree” and “Slightly disagree” were combined to form the category “Disagree”. This entails a survey result presentation with responses of agree, partially agree, disagree and not relevant. Data were analysed using SPSS version 28.0 (IBM Corp, Armonk, NY).

## Results

The survey was distributed to 1625 patients and was completed by 366 patients (23%) of which 74 (20%) were women. The average age of the patients was 69.1 ± 11.3 years. Of the 366 responses, 207 patients (57%) responded that they had used the DPE, and 159 patients (43%) responded that they had never used the DPE.

### **Non-users of the digital patient education programme**

**Fig. 1:**
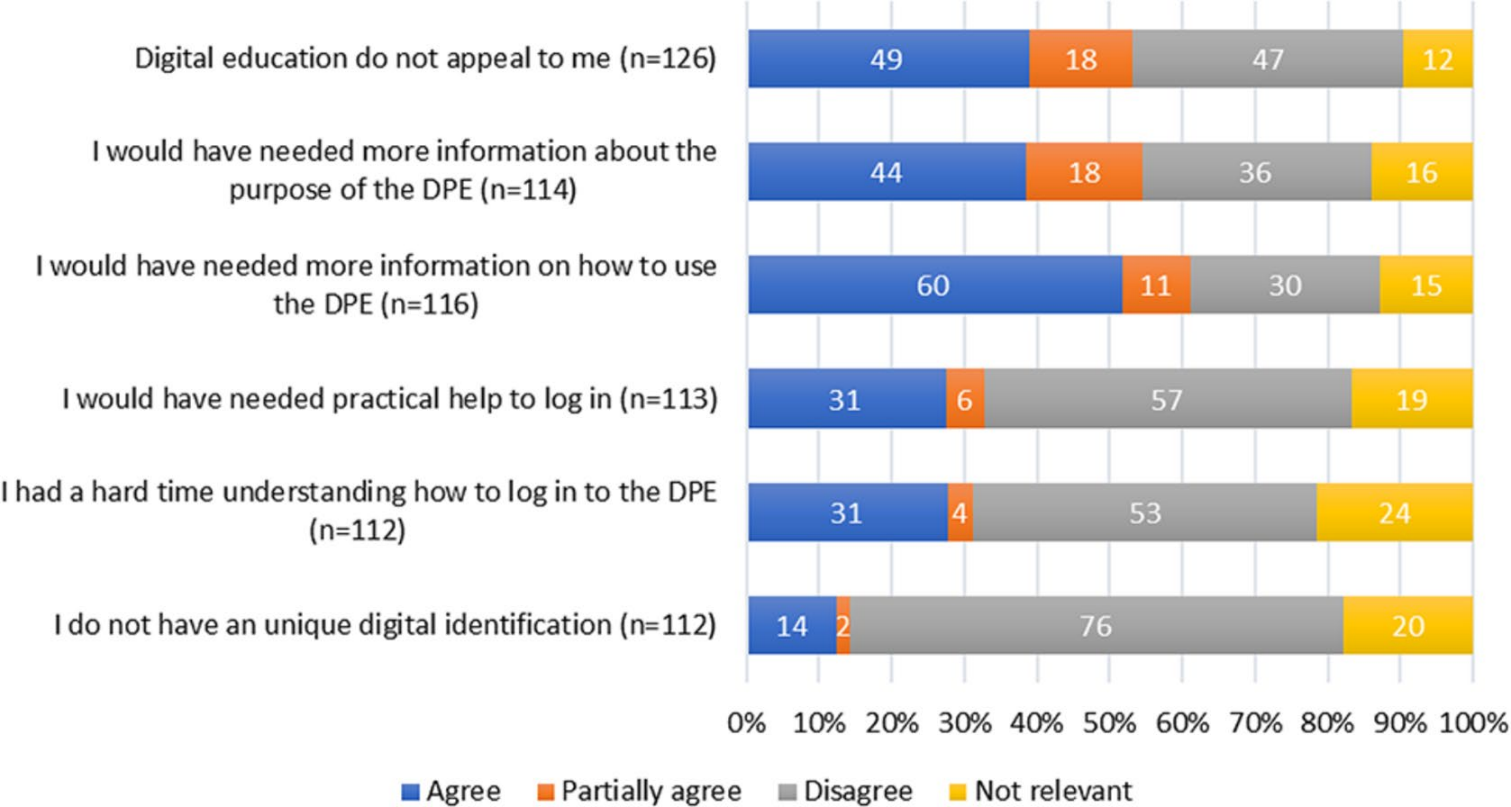
Presentation of the predetermined reasons for not using the digital patient education

**Fig. 2:**
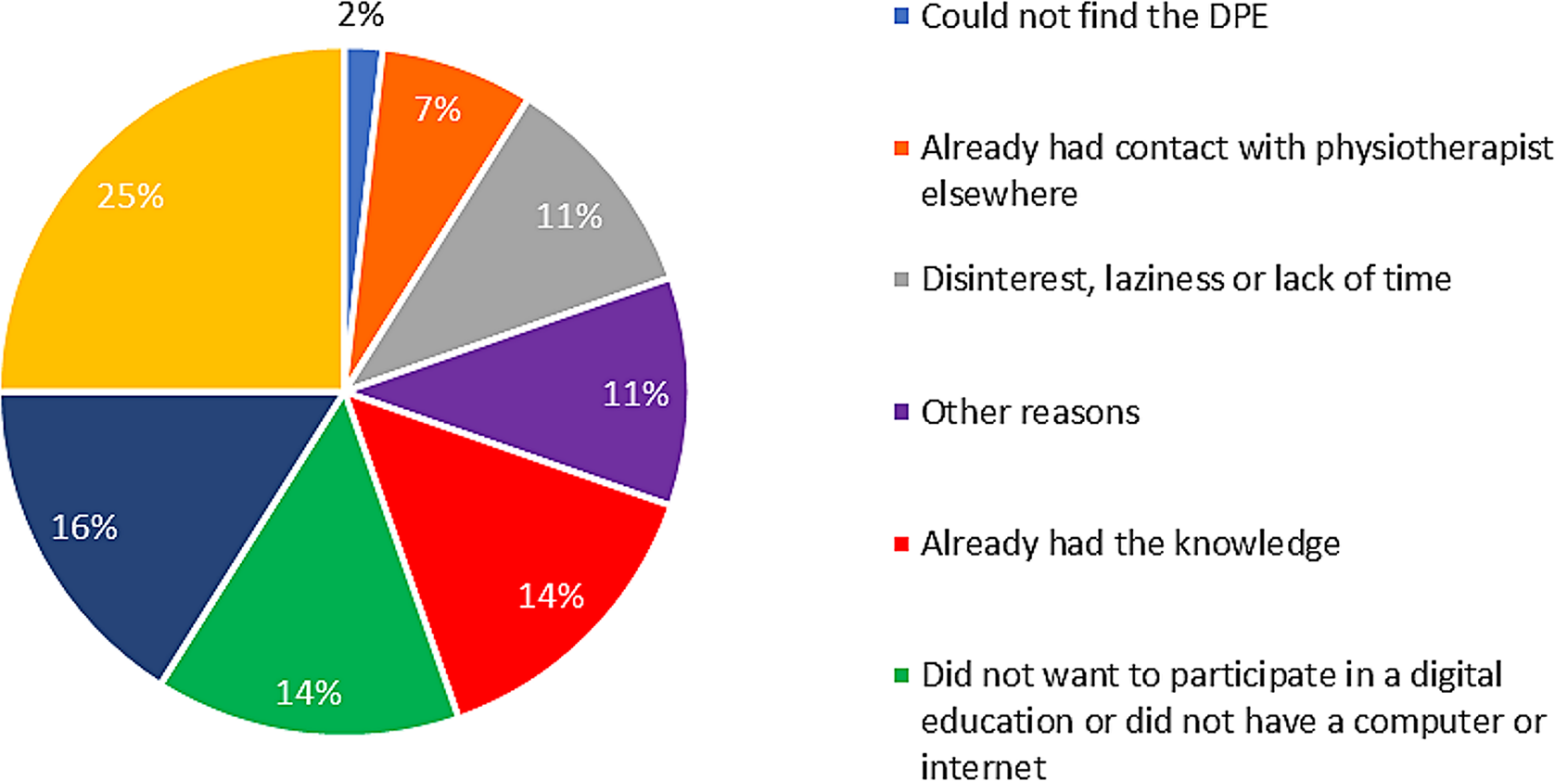
Presentation of categorised reasons given in free text for not using the digital patient education. The category “other reasons” include, i.e. visual impairment, moving to another city or not feeling well

The average age among the 159 non-users was 71.4 ± 10.6 years of which 29 patients (18%) were women. The most common reasons for not using the DPE were a perceived need of more information about how to use the DPE, *n* = 60 (52%), digital education was not found appealing, *n* = 49 (39%) and the need for more information about the purpose of the DPE, *n* = 44 (39%). On the other hand, 14 patients (13%) stated that not having a unique digital identification was a major reason for not using the DPE. The detailed survey result of reasons for not using the DPE are presented in Fig. 1. Figure 2 presents additional reasons (*n* = 56), given in free text by the non-users. The most common reasons, given in free text, for not using the DPE were that the patients did not know about the DPE, *n* = 14 (25%) and that the patients forgot to participate in the DPE, *n* = 8 (16%).

### Users of the digital patient education programme

The average age of the 207 patients who used the DPE was 67.3 ± 11.6 years and 46 patients (22%) were women.

### Perceived acceptance and usability

#### Patient’s acceptance

The survey results showed that 70 patients (35%) would have preferred to take part in a DPE at the hospital, 51 patients (26%) would have preferred the DPE as a group session and 70 patients (35%) would have preferred to participate in a DPE in real time with the possibility to ask questions to the healthcare providers (Table 1).

### Usefulness

Patients stated that the DPE improved their access to healthcare services due to being able to use the DPE whenever it suited them, *n* = 150 (75%) and due to being able to use the DPE on several occasions, *n* = 132 (67%). In addition, 102 patients (52%) found that the DPE saved time by not having to travel to the hospital and 134 patients (69%) found that the DPE provided their healthcare needs (Table 1).

### Ease of use and learnability

In total, 159 patients (80%) found the DPE simple to use and 161 patients (81%) found the DPE easy to learn to use (Table 1).

### Interface quality

As shown in Table 1, most patients, *n* = 173 (90%) agreed that the DPE was simple and easy to understand, 100 patients (51%) liked using the DPE, and 127 patients (64%) found that the DPE did everything they would want it to do.

### Interaction Quality

For 137 patients (70%) the written information was a good support, and 131 patients (66%) perceived the short video clips to be a good support, and 149 patients (76%) found the scope of the DPE satisfying (Table 1).

### Reliability

Eighty-six patients (43%) believed that the DPE was equivalent to an in-person patient education programme (Table 1).

### Satisfaction and future use

The majority of patients, *n* = 140 (70%) would use the DPE again, 127 patients (64%) accepted the DPE as a way of receiving healthcare services and 137 patients (69%) were generally satisfied with the DPE (Table 1).

**Table 1:** Patient’s acceptance and usability factors of the digital patient education

Survey questions	Agree	Partially agree	Disagree	Not relevant
**Patient’s acceptance of using the DPE, n (%)**				
I would have preferred to take part in a DPE at the hospital	70 (35)	29 (15)	92 (46)	7 (4)
I would have preferred the DPE as a group session	51 (26)	41 (21)	99 (50)	7 (4)
I would have preferred to participate in a DPE in real time with the possibility to ask questions to the healthcare providers	70 (35)	50 (25)	71 (36)	7 (4)
**Usefulness**,** n (%)**				
The DPE improved my access to healthcare services because I could take part when it suited me	150 (75)	23 (12)	17 (9)	10 (5)
The DPE improved my access to healthcare services because I could take part on several occasions	132 (67)	36 (18)	19 (10)	11 (6)
The DPE saved me time traveling to a hospital	102 (52)	40 (20)	35 (20)	20 (10)
The DPE provided my healthcare needs	134 (69)	33 (17)	22 (11)	6 (3)
**Ease of use and learnability**,** n (%)**				
It was simple to use the DPE	159 (80)	26 (13)	9 (5)	4 (2)
It was easy to learn to use the DPE	161 (81)	27 (14)	8 (4)	4 (2)
**Interface Quality**,** n (%)**				
I liked using the DPE	100 (51)	53 (27)	32 (16)	13 (7)
The DPE did everything I would want it to be able to do	127 (64)	43 (22)	19 (10)	9 (5)
The DPE was simple and easy to understand	173 (90)	8 (4)	6 (3)	6 (3)
**Interaction Quality**,** n (%)**				
The written information was a good support	137 (70)	42 (22)	10 (5)	6 (3)
The short video clips were a good support	131 (66)	29 (15)	20 (10)	17 (9)
The scope of the DPE was satisfying	149 (76)	25 (13)	15 (8)	6 (3)
**Reliability**,** n (%)**				
I think the DPE is the same as an in-person patient education	86 (43)	59 (30)	47 (24)	8 (4)
**Satisfaction and future use**,** n (%)**				
I would use the DPE services again	140 (70)	27 (14)	24 (12)	9 (5)
The DPE was an acceptable way to receive healthcare services	127 (64)	50 (25)	17 (9)	5 (3)
In general, I am satisfied with the DPE	137 (69)	39 (19)	18 (9)	6 (3)

Variables are presented as number (%). DPE, Digital patient education

### Digital patient education modules of coronary artery disease pharmacological treatment,  exercise based cardiac rehabilitation and risk factors

**Fig. 3:**
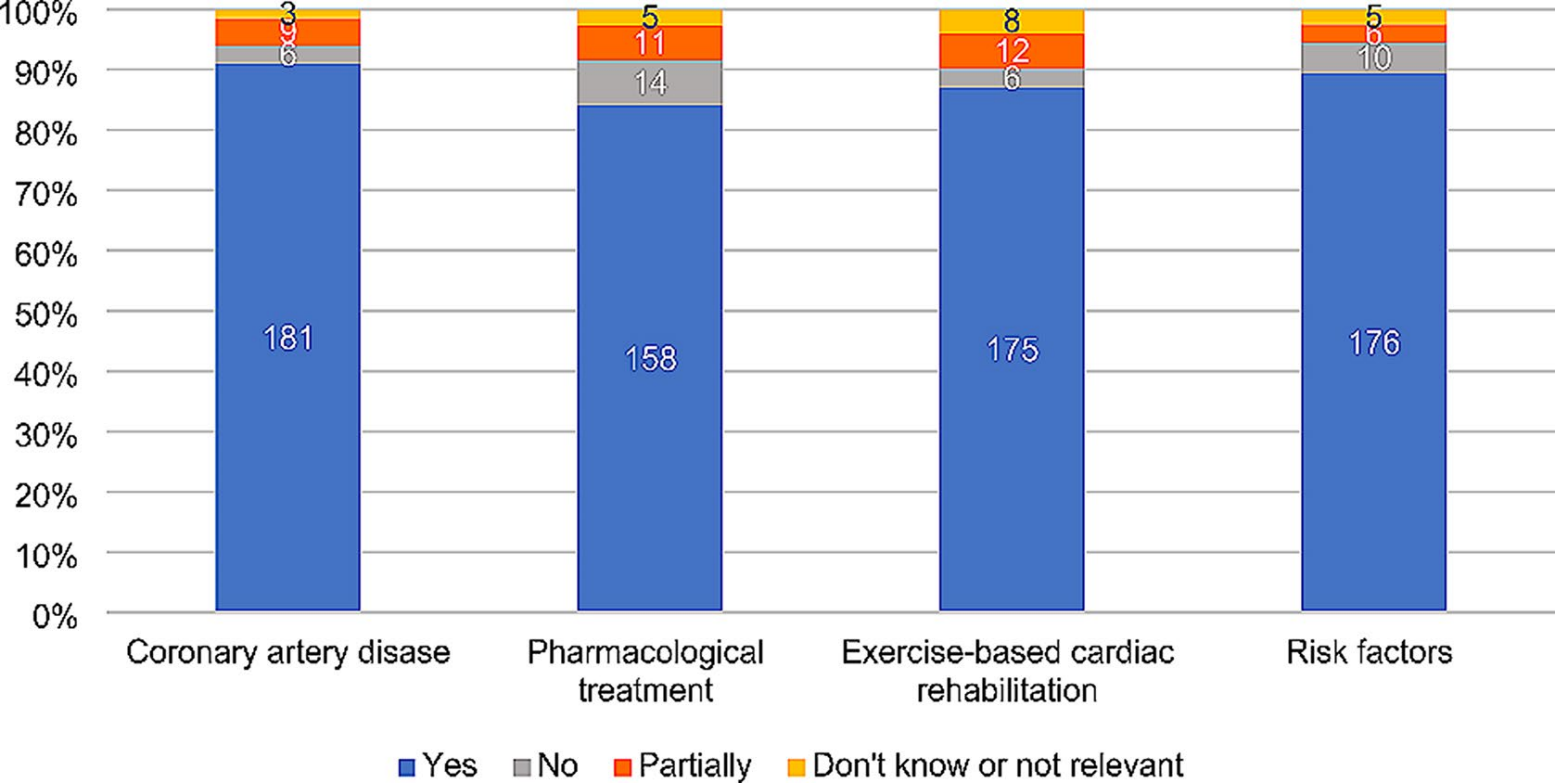
Presentation of patients completing the digital patient educational modules about coronary artery disease, pharmacological treatment, exercise-based cardiac rehabilitation, and risk factors

The survey results showed that > 80% of the patients completed the DPE modules about CAD, pharmacological treatment, exercise-based cardiac rehabilitation and risk factors (Figs. 3) and 81–90% of the patients found these modules interesting (details not shown).

### Digital patient education modules about healthy lifestyle recommendations

**Fig. 4:**
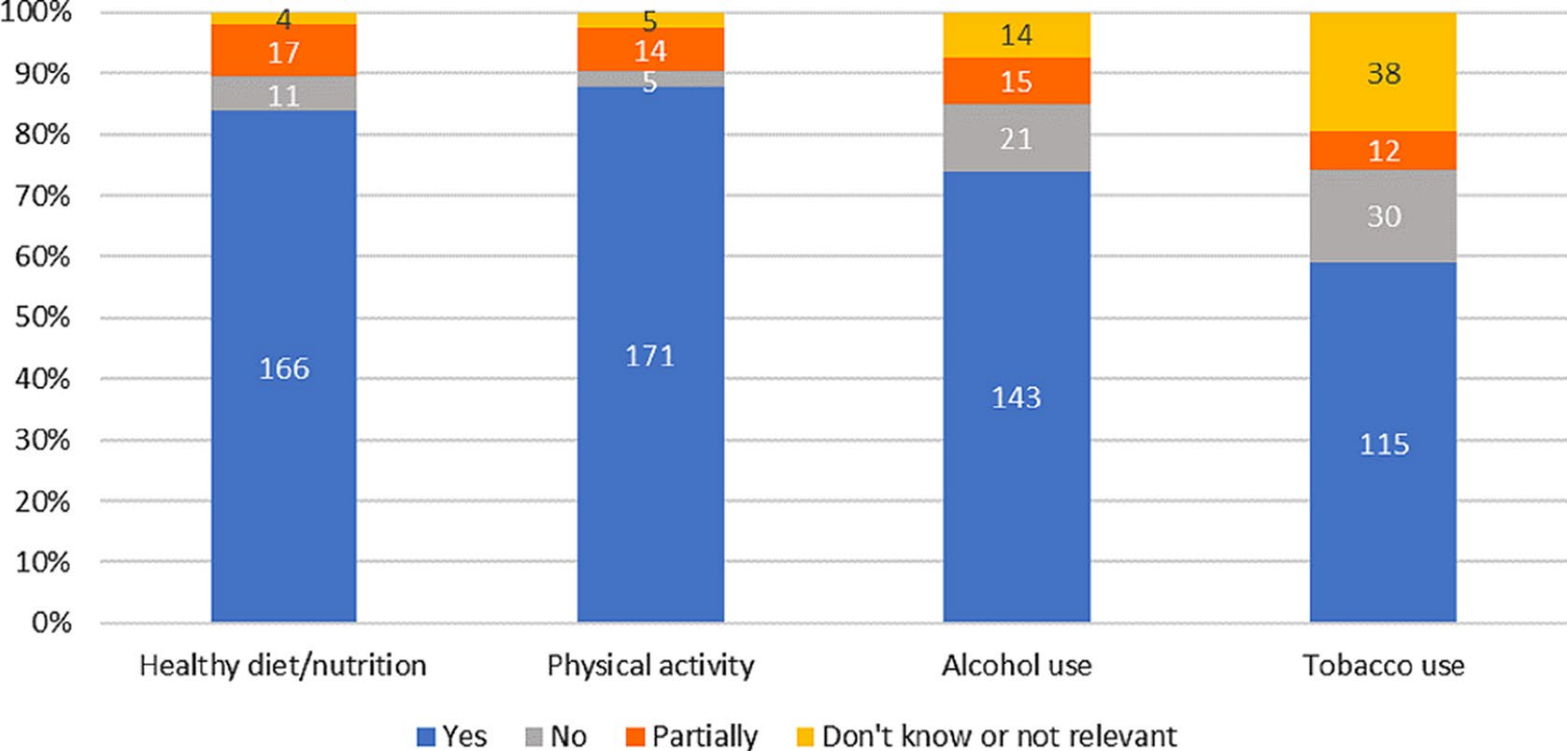
Presentation of patients completing the digital patient educational modules about healthy lifestyle recommendations

As seen in Figs. 4 and 166 patients (84%) completed the DPE module about healthy diet/nutrition and 171 patients (88%) completed the module about physical activity. A total of 82% of the patients found the module healthy diet/nutrition interesting and 87% found the physical activity module interesting (details not shown). The corresponding result for completing the modules about alcohol and tobacco use were 143 patients (74%) and 155 patients (59%), respectively (Fig. 4). Additionally, 65% of the patients found the alcohol use module interesting and 58% of the patients found the tobacco use module interesting (details not shown).

### Digital patient education modules of emotional reactions, follow-up visit at the cardiac rehabilitation outpatient clinic and the Heart- and Lung Association

There were 142 patients (76%) who completed the module of emotional reactions and 70% of the patients found the module interesting. A total of 127 patients (66%) completed the module follow-up visit at the cardiac rehabilitation outpatient clinic and 81% of the patients agreed that this module was interesting. For the module Heart- and Lung Association, 101 patients (54%) completed the module and 43% of the patients found it interesting (details not shown).

### Knowledge of secondary prevention and healthy lifestyle

**Fig. 5:**
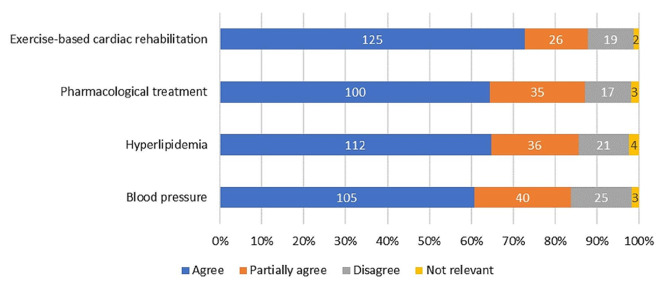
Proportion of patients who found that the digital patient education had increased their knowledge about treatment goal regarding blood pressure, hyperlipidaemia, pharmacological treatment and exercise-based cardiac rehabilitation

**Fig. 6:**
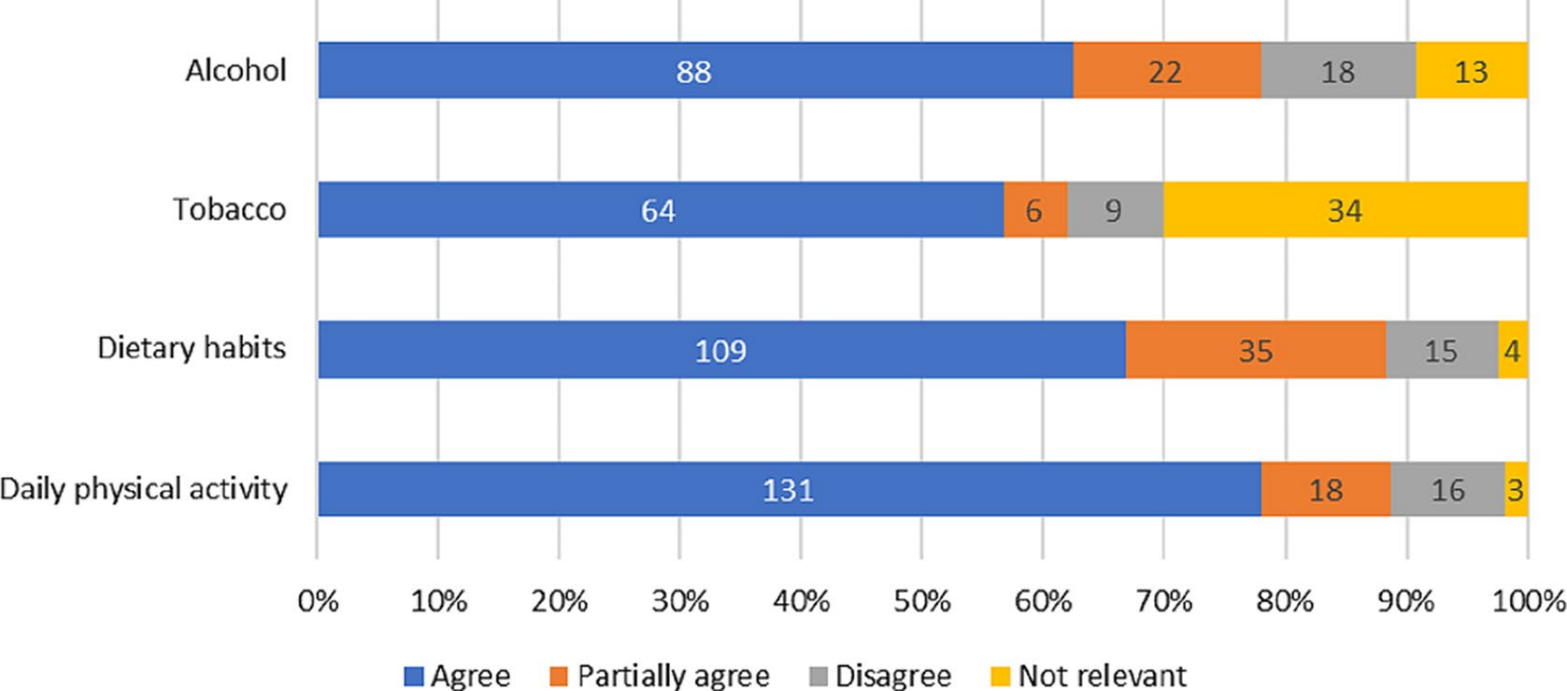
Proportions of patients who found that the digital patient education had increased their knowledge about factors of a healthy lifestyle including daily physical activity, dietary habits, use of tobacco and alcohol

As seen in Fig. 5, > 60% of the patients agreed that the DPE increased their knowledge about treatment goals for blood pressure, hyperlipidaemia, pharmacological treatment, and exercise based cardiac rehabilitation. Regarding healthy lifestyle recommendations, 131 patients (78%) found that the DPE increased their knowledge about daily physical activity and, 109 patients (67%) agreed that the DPE increased their knowledge about recommended healthy food choices. Corresponding results for increased knowledge about alcohol use was 88 patients (62%) and 64 patients (57%) for tobacco use. However, 34 patients (30%) responded that information about tobacco and smoking cessation was irrelevant (Fig. 6).

### Motivation of making healthy lifestyle changes and having a healthy lifestyle today

**Fig. 7:**
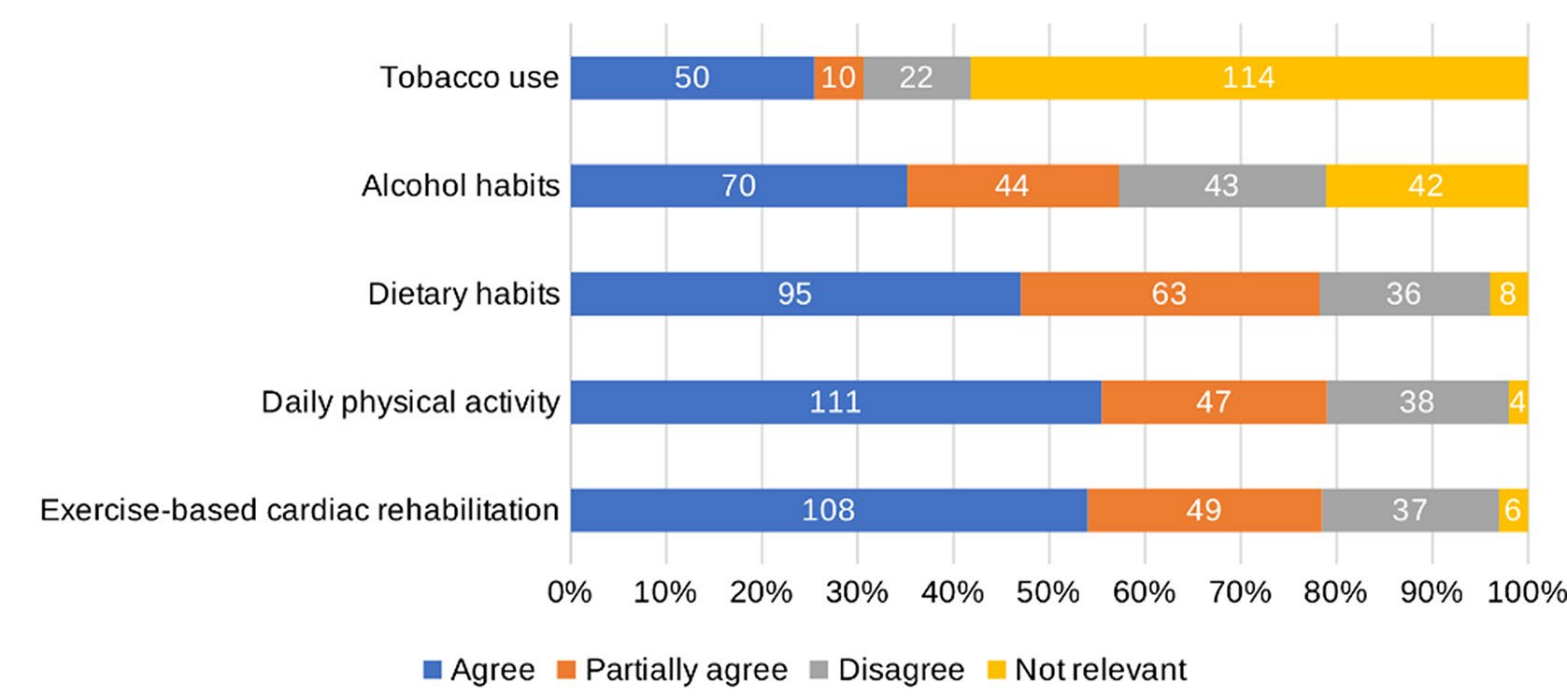
Proportion of patients who agreed that the digital patient education had motivated them to make significant healthy lifestyle changes regarding exercise-based cardiac rehabilitation, daily physical activity, dietary habits, alcohol habits and tobacco use

**Fig. 8:**
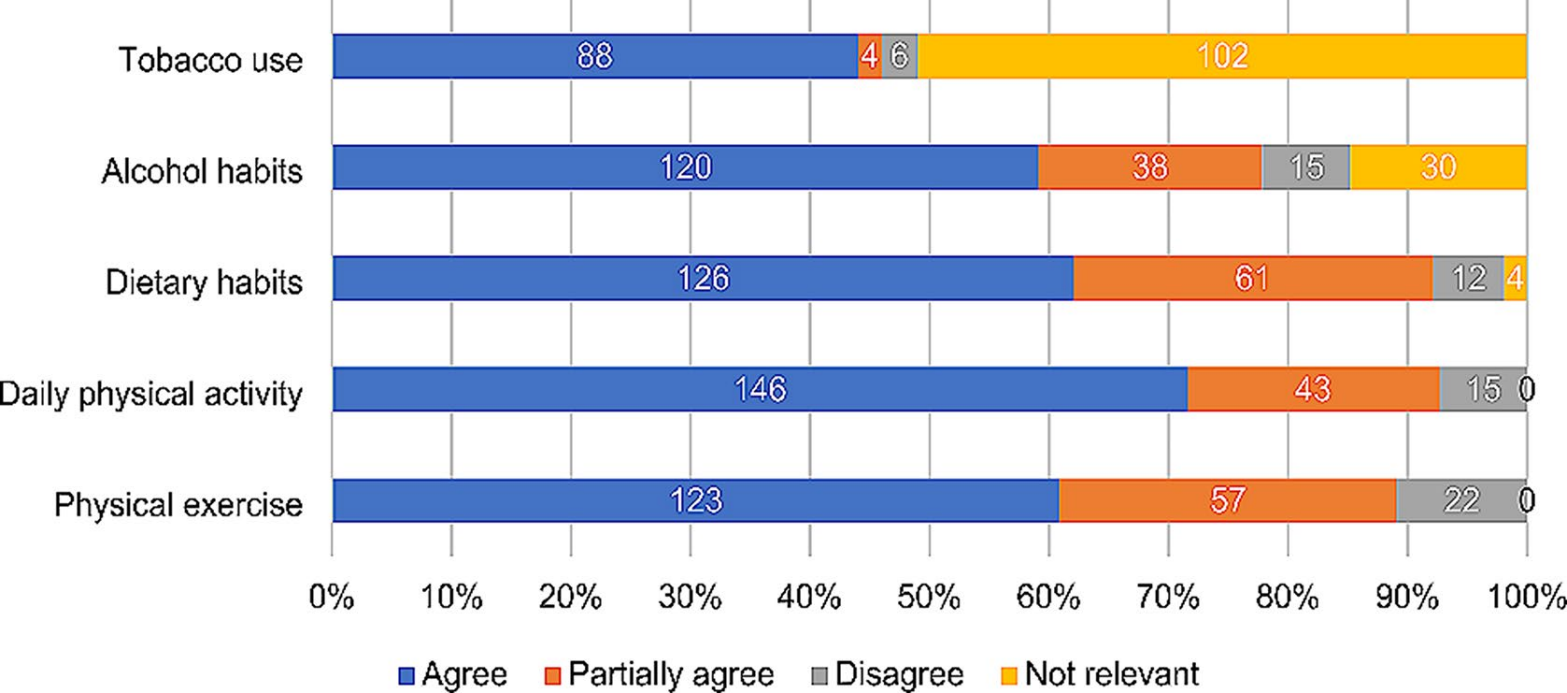
Proportion of patients who report to have a healthy lifestyle regarding physical exercise, daily physical activity, dietary habits, alcohol habits and tobacco use

The survey results show that approximately 55% of the patients agreed that the DPE motivated them to make healthy lifestyle changes in terms of daily physical activity and participation in exercise based cardiac rehabilitation (Fig. 7). The corresponding results of healthy dietary habits was 95 patients (47%) and for alcohol use, 70 patients (35%). Regarding tobacco use, 50 patients (26%) found the DPE motivating to change behaviour, but 114 patients (59%) found the item of tobacco use irrelevant (Fig. 7). Figure 8 shows that about 60% of the patients agreed to have a healthy lifestyle today regarding exercise, *n* = 123 (61%), daily physical activity, *n* = 146 (72%), dietary habits, *n* = 126 (62%) and alcohol habits, *n* = 120 (59%). The corresponding result for tobacco use was 88 patients (44%), but 102 patients (51%) found tobacco use irrelevant (Fig. 8).

### Additional experiences and opinions about digital patient education

In total, 90 patients (44%) gave 130 comments altogether in free text with additional experiences and opinions about the DPE. The comments were sorted into seven categories of responses. Most comments, *n* = 33 (25%) concerned ideas or reflections for improvement of the DPE. All categories with examples of comments are presented in Table 2.

**Table 2:** Free text comments of additional experiences and opinions about the digital patient education, presented by category

Category	Number of comments (%)	Examples of comments
Ideas or reflections for improvement of the DPE	33 (25)	Patients suggested that the DPE should not have time limited period of use and to add a reminder function. Patients suggested to combine the DPE with in-person education.
Positive feedback for the DPE	26 (20)	Patients found the DPE to be good and well developed. Patients found it positive not to have scheduled visits with a clinic.
Comments related to disease knowledge and treatment options	23 (18)	A few patients found the knowledge in the DPE superficial, some patients would like wider or deeper knowledge.
Criticism of the DPE	19 (15)	Some patients experienced that the DPE assumed that one had poor lifestyle habits and that the DPE did not inform about what to do if you already have a healthy lifestyle.
Comments about the timing of the survey	12 (15)	Patients thought that the DPE evaluation survey was sent a long time after they had participated in the DPE, and found it difficult to remember details.
Not used the DPE	11 (8)	Patients commented that they had not used the DPE.
Digital related comments	6 (5)	A few patients had difficulty with the digital technology or did not appreciate having to read digitally.

Comments are presented by numbers and (%). DPE, digital patient education

## Discussion

This survey-based study provides increased knowledge of patients’ perceptions of a DPE as part of CR, in terms of user acceptance and usability, perceived significance on healthy lifestyle changes and secondary preventive goal fulfilment in patients after having suffered an acute CAD. Digital health systems are progressing rapidly across all fields of medicine, including cardiology [17]. In our study, the DPE was delivered on a web-based platform and included written information, short video clips and illustrations. Of the patients using the DPE, almost (69%) were generally satisfied with the DPE, and the majority considered the DPE to be simple to use and easy to learn to use and 70% of the patients would consider using it again. These results support the long-term role and continued development of the DPE. By assessing and evaluating the usability of digital health systems, the effectiveness of both the technology and service/s offered can be improved [20] and usability evaluation is also important for a successful implementation of those systems [21]. However, digital health systems may not suit all patients. Our results indicated that some patients preferred the DPE while some patients would rather have participated in the centre-based patient education programme, where you can also interact with health care professionals and other persons with CAD. A previous systematic review showed that secondary preventive e-Health programmes were mainly offered to patients with CAD who could either not attend centre-based CR or as an adjunct, rather than completely replacing centre-based patient education [15]. From a person-centred perspective, it could be beneficial for accessibility to patient education in the future to offer patients both a centre-based and a digital patient education programme, or a hybrid of both, so that patients have the ability to choose the setting themselves. More research is needed to develop and evaluate the effectiveness of these different setting options.

One aspect that may affect a patient’s ability to initiate or maintain positive health behaviours is health literacy. According to WHO, health literacy refers to the personal characteristics and social resources needed for individuals and communities to access, understand, appraise and use information and services to make decisions about health [22]. Low health literacy levels have shown to be particularly common in individuals with cardiovascular diseases [23] and a study that evaluated CR programs and health literacy levels showed that more than 60% of patients with CAD had low levels of health literacy [24]. Moreover, low health literacy is associated with poorer general health status and an increased probability of rehospitalisation and mortality [14]. Health literacy may influence many aspects of our study. Our survey results of accessing and using the DPE, showed that the majority (57%) of the patients had used the DPE, although a significant percentage (43%) of the patients had not used the DPE. This result can be compared with a nationwide registry study showing that only 37% of the patients with first-time myocardial infarction attend patient education as a part of centre-based CR [25]. When implementing a DPE, also eHealth literacy is important to consider, which means that patients need the skills to find, understand, critically appraise and use health information from eHealth resources [26]. A study by Melholt et al. [27] showed that cardiac telerehabilitation via a web portal improved the patients’ skills in eHealth literacy. The authors suggest that besides managing their heart disease, the patients also became more confident in using information online [27] which is positive in today’s society where a lot of information is put online.

Reasons for not using the DPE are multifaceted and need to be discussed in the light of further development and implementation of the DPE. Firstly, our results indicated that the non-users of the DPE were on average four years older than the patients using the DPE. A previous systematic review demonstrated that higher age was a predictive factor of non-participation in cardiac telerehabilitation programmes [28]. This systematic review also included additional predictive factors for non-participation, such as lower education level and current smoking, which was not part of the data collection in our study [28]. Secondly, our study showed that patients would have needed more information about how to use and the purpose of using the DPE. It may be hypothesised that this finding is related to the short hospital stay after an acute coronary event and that the patient receives a lot of information during this hospital admission. In the future, to increase the numbers of DPE users, adjustments to factors such as age should be explored, and healthcare professionals need to improve their delivery strategies for patient information about the DPE during admission to hospital. Moreover, previous research has shown the potential importance of email reminders and patient education about how to use a web-based intervention of lifestyle changes in patients with coronary heart disease or chronic back pain [29]. These suggestions may also be a way forward to increase the number of patients using the DPE in the future.

Furthermore, health literacy includes understanding and using health information [22]. In our study, > 60% of the patients found that the DPE increased their knowledge about treatment goals for blood pressure, hyperlipidaemia, pharmacological treatment, and exercise based cardiac rehabilitation. These findings are in line with the results from an observational study by Williamsson et al. [30]. Their study objective was to evaluate 12 weeks of center-based CR including structured patient education in patients with CAD by assessing patients CAD-related knowledge at three times; before and after 12 weeks and at 3 months follow-up. CAD knowledge was measured using the Coronary Artery Disease Education Questionnaire-2nd Version (CADE-Q-II). The authors concluded that patients with CAD who received patient education within CR showed improvements in disease-related knowledge that persisted at 3-month follow-up [30]. However, our study results of increased knowledge about the treatment goals of secondary disease prevention are based on the patients’ perceptions of increased knowledge and not an assessment of their actual knowledge. Integrating some kind of quiz or questionnaire in the DPE for patients to evaluate their own knowledge and for healthcare professionals to be able to assess patient knowledge may be a part of future DPE development.

The promotion of adherence to pharmacologic therapies and lifestyle recommendations is an important part of cardiac rehabilitation programmes [7]. However, making and maintaining lifestyle changes can be challenging. About 60% of the patients in our study reported having a healthy lifestyle today regarding exercise, daily physical activity, dietary habits, and alcohol use and, the majority of the patients were motivated by the DPE to make healthy lifestyle changes with regards to physical activity and exercise-based cardiac rehabilitation. These findings are in line with a systematic review by Ghisi et al. [31], showing that educational interventions (individual or group-based) as part of CR was associated with increased levels of physical activity, healthier dietary habits, and smoking cessation. However, any related improvement in response to cardiac symptoms, medication compliance or psychosocial well-being were more uncertain [31]. In addition, a systematic review by Shi et al. [32] demonstrated that patient education about secondary prevention more than doubled the probability of adherence to physical activity guidelines at < 6 months and 6–12 months follow up in patients with coronary heart disease. The patients were three times more likely to adhere to healthy dietary advice at < 6 months and more likely to sustain this healthy behaviour at 6–12 months follow up when participated in educational interventions for secondary prevention [32]. Furthermore, a Swedish nationwide registry study from 2019 demonstrated that participating in a centre-based patient education programme, as part of CR, was associated with 50% reduction of all-cause and CV mortality in patients with first-time myocardial infarction [25]. Associations between participation in a DPE and similar clinical outcomes should be assessed in future studies.

### Strengths and limitations

One strength in this study was that patients could choose to answer the survey on paper or digitally. Web-based surveys are shown to have a significantly lower response rate than paper-based surveys but on the other hand, web-based data collection has a considerable cost advantage compared with paper-based data collection [33]. The survey response rate in our study was 23%, which can be compared with a cross-sectional study from Switzerland that evaluated patient hospital satisfaction with a response rate ranging from 16.1 to 80.0% [34]. In addition, a survey-based study exploring patients’ opinions regarding satisfaction with telemedicine-provided care had a response rate of 5.8% [35]. Suggested strategies as the use of e-mail pre-notification, email invitation and two reminders are examples of methods for increasing the response rates in web-based surveys [36]. Another strength was the inclusion of open-ended questions, which provided additional information on patients’ experience of the DPE. To avoid sampling bias, all patients with CAD who were registered users of the DPE were given the opportunity to participate in the study. Furthermore, the DPE was developed in co-design with patients to meet end users´ information and support needs which may enhance usability.

The current study is not without limitations. As all surveys were sent at the same time point, the time between participating and evaluating the DPE differs among the patients. A longer time interval between participation and filling out the survey may have affected patients’ perceptions of the DPE as well as a potential recall bias. No demographic patient information was collected in this study. Data such as level of education, occupational status, or travel distance to the hospital, could have enable deeper result analysis. In addition, the survey’s omission of questions regarding patients’ tobacco use restricts additional analysis on this topic.

## Conclusions

This study adds new knowledge about a digital patient education programme (DPE), as part of CR, in patients with acute CAD. The survey results demonstrate overall high levels of patient acceptance and usability of the DPE, and this supports its long-term role and continued development. The majority of respondents reported that the DPE increased their knowledge about secondary preventive treatment goals and most of the patients reported having a healthy lifestyle today, which is important to reduce the risk of recurrent coronary events. Associations between participation in the DPE and clinical outcomes must however be further evaluated in future studies.

### Electronic supplementary material

Below is the link to the electronic supplementary material.

Supplementary Material 1

## Data Availability

Availability of data and materials: Informed consent was not obtained for publication of patient data. Upon reasonable request, deidentified data may be available from the corresponding author.

## Abbreviations

AUDIT: Alcohol Use Disorders Identification Test
CR: Cardiac rehabilitation
CAD: Coronary artery disease
DPE: Digital patient education programme
TUQ: Telehealth usability questionnaire
