# Supplementary material for: Evaluation of a digital patient education programme in patients with coronary artery disease, a survey-based study
Source: BMC Health Serv Res. 2024 Sep 2;24:1012. doi: 10.1186/s12913-024-11374-5 (PMC11370250; doi:10.1186/s12913-024-11374-5)
Supplement: Supplementary file 1 — Supplementary Material 1 [file 12913_2024_11374_MOESM1_ESM.docx]

**Evaluation of a digital patient education programme in patients with
coronary artery disease**

1. Do you have logged in to the digital patient education programme?
   - Yes, continue to question 3
   - No, please answer only question 2
2. If you have not logged in to the digital patient education programme, please answer the following statements:

|  | Completely agree | Strongly agree | Partially agree | Slightly disagree | Completely disagree | Not relevant |
| --- | --- | --- | --- | --- | --- | --- |
| I do not have a unique digital identification | ○ | ○ | ○ | ○ | ○ | ○ |
| I had a hard time understanding how to log in to the digital patient education | ○ | ○ | ○ | ○ | ○ | ○ |
| I would have needed practical help to log in | ○ | ○ | ○ | ○ | ○ | ○ |
| I would have needed more information on how to use the digital patient education | ○ | ○ | ○ | ○ | ○ | ○ |
| I would have needed more information about the purpose of the digital patient education | ○ | ○ | ○ | ○ | ○ | ○ |
| Other reason | ○ | ○ | ○ | ○ | ○ | ○ |

1. If you have logged in to the digital patient education programme, which modules have you taking part of?

|  | Yes | No | Partially | Don’t know | Not relevant |
| --- | --- | --- | --- | --- | --- |
| Coronary artery disease | ○ | ○ | ○ | ○ | ○ |
| Risk factors | ○ | ○ | ○ | ○ | ○ |
| Follow-up visit at the cardiac rehabilitation outpatient clinic | ○ | ○ | ○ | ○ | ○ |
| Pharmacological treatment | ○ | ○ | ○ | ○ | ○ |
| Exercise-based cardiac rehabilitation | ○ | ○ | ○ | ○ | ○ |
| Physical activity | ○ | ○ | ○ | ○ | ○ |
| Healthy diet/nutrition | ○ | ○ | ○ | ○ | ○ |
| Tobacco | ○ | ○ | ○ | ○ | ○ |
| Alcohol | ○ | ○ | ○ | ○ | ○ |
| Emotional reactions | ○ | ○ | ○ | ○ | ○ |
| Heart- and Lung Association | ○ | ○ | ○ | ○ | ○ |

1. Which modules in the digital patient education programme did you find interesting? Choose the option that suits you best for each module.

|  | Completely agree | Strongly agree | Partially agree | Slightly disagree | Completely disagree | Not relevant |
| --- | --- | --- | --- | --- | --- | --- |
| Coronary artery disease | ○ | ○ | ○ | ○ | ○ | ○ |
| Risk factors | ○ | ○ | ○ | ○ | ○ | ○ |
| Follow-up visit at the cardiac rehabilitation outpatient clinic | ○ | ○ | ○ | ○ | ○ | ○ |
| Pharmacological treatment | ○ | ○ | ○ | ○ | ○ | ○ |
| Exercise-based cardiac rehabilitation | ○ | ○ | ○ | ○ | ○ | ○ |
| Physical activity | ○ | ○ | ○ | ○ | ○ | ○ |
| Healthy diet/nutrition | ○ | ○ | ○ | ○ | ○ | ○ |
| Tobacco | ○ | ○ | ○ | ○ | ○ | ○ |
| Alcohol | ○ | ○ | ○ | ○ | ○ | ○ |
| Emotional reactions | ○ | ○ | ○ | ○ | ○ | ○ |
| Heart- and Lung Association | ○ | ○ | ○ | ○ | ○ | ○ |

1. Here are several statements about the structure of the content in the digital patient education programme. Respond to the statement with the option that you find correct.

|  | Completely agree | Strongly agree | Partially agree | Slightly disagree | Completely disagree | Not relevant |
| --- | --- | --- | --- | --- | --- | --- |
| The written information was a good support | ○ | ○ | ○ | ○ | ○ | ○ |
| The short video clips were a good support | ○ | ○ | ○ | ○ | ○ | ○ |
| It was simple to use the digital patient education | ○ | ○ | ○ | ○ | ○ | ○ |
| The scope of the digital patient education was satisfying | ○ | ○ | ○ | ○ | ○ | ○ |
| The digital patient education provided my healthcare needs | ○ | ○ | ○ | ○ | ○ | ○ |

1. Did you take part in the digital patient education programme together with a relative?
   - Yes
   - No
2. Did you use the message function?
   - Yes
   - No, continue with question 10
3. Are you satisfied with the message function?
   - Yes
   - No
   - Partially
4. Did you get a response in a reasonable time?
   - Yes
   - No
5. Here are several statements about the digital patient education programme. Respond to the statement with the option that you find correct.

|  | Completely agree | Strongly agree | Partially agree | Slightly disagree | Completely disagree | Not relevant |
| --- | --- | --- | --- | --- | --- | --- |
| It was easy to learn to use the digital patient education | ○ | ○ | ○ | ○ | ○ | ○ |
| The digital patient education improved my access to healthcare services because I could take part on several occasions | ○ | ○ | ○ | ○ | ○ | ○ |
| The digital patient education improved my access to healthcare services because I could take part when it suited me | ○ | ○ | ○ | ○ | ○ | ○ |
| The digital patient education saved me time traveling to a hospital | ○ | ○ | ○ | ○ | ○ | ○ |
| I would have preferred to take part in a digital patient education at the hospital | ○ | ○ | ○ | ○ | ○ | ○ |
| I would have preferred the digital patient education as a group session | ○ | ○ | ○ | ○ | ○ | ○ |
| I would have preferred to participate in a digital patient education in real time with the possibility to ask questions to the healthcare providers | ○ | ○ | ○ | ○ | ○ | ○ |
| It was simple to use the digital patient education | ○ | ○ | ○ | ○ | ○ | ○ |
| I liked using the digital patient education | ○ | ○ | ○ | ○ | ○ | ○ |
| The digital patient education did everything I would want it to be able to do | ○ | ○ | ○ | ○ | ○ | ○ |
| The digital patient education was an acceptable way to receive healthcare services | ○ | ○ | ○ | ○ | ○ | ○ |
| I would use the digital patient education services again | ○ | ○ | ○ | ○ | ○ | ○ |
| In general, I am satisfied with the digital patient education | ○ | ○ | ○ | ○ | ○ | ○ |

1. The digital patient education has increased my knowledge about treatment goals for coronary artery disease regarding:

|  | Completely agree | Strongly agree | Partially agree | Slightly disagree | Completely disagree | Not relevant |
| --- | --- | --- | --- | --- | --- | --- |
| Blood pressure | ○ | ○ | ○ | ○ | ○ | ○ |
| Hyperlipidaemia | ○ | ○ | ○ | ○ | ○ | ○ |
| Pharmacological treatment | ○ | ○ | ○ | ○ | ○ | ○ |

1. The digital patient education has increased my knowledge about factors of having a healthy lifestyle in coronary artery disease regarding:

|  | Completely agree | Strongly agree | Partially agree | Slightly disagree | Completely disagree | Not relevant |
| --- | --- | --- | --- | --- | --- | --- |
| Exercise-based cardiac rehabilitation | ○ | ○ | ○ | ○ | ○ | ○ |
| Daily physical activity | ○ | ○ | ○ | ○ | ○ | ○ |
| Dietary habits | ○ | ○ | ○ | ○ | ○ | ○ |
| Alcohol | ○ | ○ | ○ | ○ | ○ | ○ |
| Tobacco | ○ | ○ | ○ | ○ | ○ | ○ |

1. The digital patient education has motivated me to make healthy lifestyle changes regarding:

|  | Completely agree | Strongly agree | Partially agree | Slightly disagree | Completely disagree | Not relevant |
| --- | --- | --- | --- | --- | --- | --- |
| Exercise-based cardiac rehabilitation | ○ | ○ | ○ | ○ | ○ | ○ |
| Daily physical activity | ○ | ○ | ○ | ○ | ○ | ○ |
| Dietary habits | ○ | ○ | ○ | ○ | ○ | ○ |
| Alcohol | ○ | ○ | ○ | ○ | ○ | ○ |
| Tobacco | ○ | ○ | ○ | ○ | ○ | ○ |

1. Today, I have a healthy lifestyle regarding:

|  | Completely agree | Strongly agree | Partially agree | Slightly disagree | Completely disagree | Not relevant |
| --- | --- | --- | --- | --- | --- | --- |
| Exercise-based cardiac rehabilitation | ○ | ○ | ○ | ○ | ○ | ○ |
| Daily physical activity | ○ | ○ | ○ | ○ | ○ | ○ |
| Dietary habits | ○ | ○ | ○ | ○ | ○ | ○ |
| Alcohol | ○ | ○ | ○ | ○ | ○ | ○ |
| Tobacco | ○ | ○ | ○ | ○ | ○ | ○ |

1. Please provide us with any additional experiences and opinions about digital patient education.

Give your answer in free text:
